# Friedreich ataxia: metal dysmetabolism in dorsal root ganglia

**DOI:** 10.1186/2051-5960-1-26

**Published:** 2013-06-19

**Authors:** Arnulf H Koeppen, Erik C Kuntzsch, Sarah T Bjork, R Liane Ramirez, Joseph E Mazurkiewicz, Paul J Feustel

**Affiliations:** 1 Research Services, Veterans Affairs Medical Center, 113 Holland Ave, Albany, NY 12208, USA; 2 Neurology Service, Veterans Affairs Medical Center, Albany, NY 12208, USA; 3 Department of Neurology, Albany Medical College, Albany, NY 12208, USA; 4 Department of Pathology, Albany Medical College, Albany, NY 12208, USA; 5 Center for Neuropharmacology and Neuroscience, Albany Medical College, Albany, NY 12208, USA

**Keywords:** Dorsal root ganglia, Ferritin, Friedreich ataxia, Iron, Metallothionein, X-ray fluorescence, Zinc, Zip14

## Abstract

**Background:**

Friedreich ataxia (FA) causes distinctive lesions of dorsal root ganglia (DRG), including neuronal atrophy, satellite cell hyperplasia, and absorption of dying nerve cells into residual nodules. Two mechanisms may be involved: hypoplasia of DRG neurons from birth and superimposed iron (Fe)- and zinc (Zn)-mediated oxidative injury. This report presents a systematic analysis of DRG in 7 FA patients and 13 normal controls by X-ray fluorescence (XRF) of polyethylene glycol-embedded DRG; double-label confocal immunofluorescence microscopy of Zn- and Fe-related proteins; and immunohistochemistry of frataxin and the mitochondrial marker, ATP synthase F1 complex V β-polypeptide (ATP5B).

**Results:**

XRF revealed normal total Zn- and Fe-levels in the neural tissue of DRG in FA (mean ± standard deviation): Zn=5.46±2.29 μg/ml, Fe=19.99±13.26 μg/ml in FA; Zn=8.16±6.19 μg/ml, Fe=23.85±12.23 μg/ml in controls. Despite these unchanged total metal concentrations, Zn- and Fe-related proteins displayed major shifts in their cellular localization. The Zn transporter Zip14 that is normally expressed in DRG neurons and satellite cells became more prominent in hyperplastic satellite cells and residual nodules. Metallothionein 3 (MT3) stains confirmed reduction of neuronal size in FA, but MT3 expression remained low in hyperplastic satellite cells. In contrast, MT1/2 immunofluorescence was prominent in proliferating satellite cells. Neuronal ferritin immunofluorescence declined but remained strong in hyperplastic satellite cells and residual nodules. Satellite cells in FA showed a larger number of mitochondria expressing ATB5B. Frataxin immunohistochemistry in FA confirmed small neuronal sizes, irregular distribution of reaction product beneath the plasma membrane, and enhanced expression in hyperplastic satellite cells.

**Conclusions:**

The pool of total cellular Zn in normal DRG equals 124.8 μM, which is much higher than needed for the proper function of Zn *ion*-dependent proteins. It is likely that any disturbance of Zn buffering by Zip14 and MT3 causes mitochondrial damage and cell death. In contrast to Zn, sequestration of Fe in hyperplastic satellite cells may represent a protective mechanism. The changes in the cellular localization of Zn- and Fe-handling proteins suggest metal transfer from degenerating DRG neurons to activated satellite cells and connect neuronal metal dysmetabolism with the pathogenesis of the DRG lesion in FA.

## Background

Dorsal root ganglia (DRG) are a primary target of Friedreich ataxia (FA) [1,2]. The reason for this vulnerability in comparison with other neural tissues is unknown. The mutation in the vast majority of patients with FA is a homozygous guanine-adenine-adenine (GAA) trinucleotide repeat expansion in intron 1 of the *FXN* gene that causes deficiency of frataxin, a small mitochondrial protein [3]. A critical effect of low frataxin levels is impaired function of iron (Fe)-sulfur cluster-dependent proteins [4]. Little disagreement exists that frataxin deficiency affects the function of mitochondrial complexes I, II, III, aconitase, and ferrochelatase, and that frataxin-depleted cultured cells are abnormally sensitive to reactive oxygen species. The role of Fe in this sensitivity to oxidative damage has been more controversial. Proponents and opponents have offered recent reviews of the evidence for Fe [5] or against Fe [6]. While assay of total Fe in DRG of FA patients did not show an increase above normal, prominent ferritin immunofluorescence in proliferating satellite cells suggested enhanced ferritin messenger RNA (mRNA) translation in response to local Fe excess [2]. The accumulation of ferritin in satellite cells surrounding dying DRG neurons, especially in the neuron-rich subcapsular region of DRG, matched Fe-specific signals on X-ray fluorescence (XRF) “maps” [2]. An upgraded version of the XRF unit allows the operator to quantify Fe and other metals *in situ* and to exclude DRG capsule, fat tissue, and adjacent spinal roots that often contain concretions of calcium (Ca), Fe, and Zn. XRF technology recently established that the neural tissue of DRG contains substantial concentrations of Zn. The importance of Zn in DRG arises from the fact that this transition metal is similar to Fe as a potential reactant in the synthesis of hydroxyl radicals through Fenton chemistry. This paper reports *in*-*situ* quantification of total Zn and Fe, and changes in the metal-carrying proteins Zip14, metallothionein (MT) 1/2 and 3, and ferritin in the DRG of FA. It also describes the frataxin deficit in DRG at the cellular level and the role of satellite cell hyperplasia in the pathogenesis of the DRG lesion in FA.

## Results

### Autopsy specimens

This work has received approval by the Institutional Review Board of the VA Medical Center, Albany, N.Y., USA. Table 1 lists basic clinical information on 7 FA patients from whom adequate DRG samples were available for XRF and matching slide preparations. All patients had homozygous GAA trinucleotide repeat expansions. The cause of death was hypertrophic cardiomyopathy in patients 2 and 4–7. Patients 1 and 3 died from cachexia in the course of their neurological disability. Normal control DRG tissue was available from 13 persons who died from non-neurological illnesses. Mean age ± standard deviation (S.D.) was 62.5±9.1 years (range 48–77). Autopsy delay was 16.3±10.9 h in the FA cases (range 3–33) and 22±20 h in the normal control subjects (range 1–56).

**Table 1:** Basic clinical information on 7 patients with FA from whom suitable DRG tissue was available for XRF

**No**. **and sex**	**Age of onset**	**Age of death**	**Disease duration**	**GAA trinucleotide repeats**
1 F	5	25	20	1100/800
2 M	7	34	27	1114/1114
3 M	7	35	28	1000/750
4 M	9	33	24	925/925
5 M	10	24	14	1050/700
6 F	15	69	54	568/568
7 F	18	63	45	730/639
Mean±S.D.*	10.1±4.7	40.4±18.1	30.3±14.2	927±205/785±185

*S.D., standard deviation.

### Qualitative and quantitative XRF of Zn and Fe

**Figure 1:**
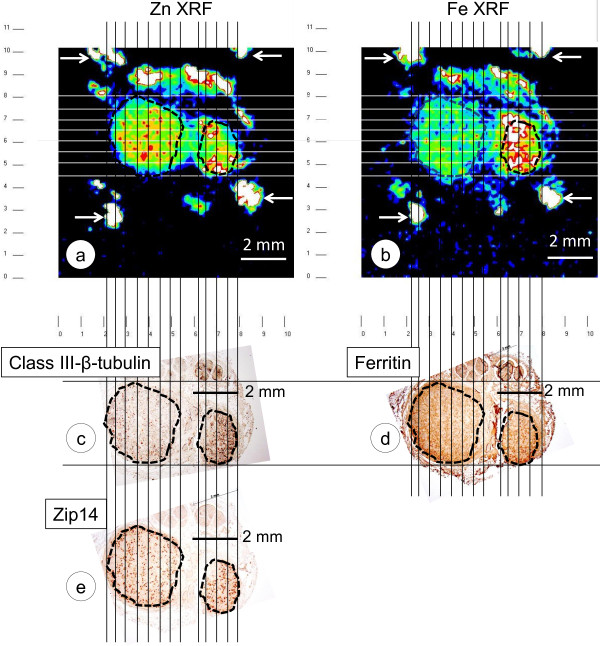
**Alignment of Zn and Fe XRF maps of a DRG in FA and matching paraffin sections.** (**a**), Zn XRF; (**b**), Fe XRF; (**c**) class-III-β-tubulin immunohistochemistry; (**d**), ferritin immunohistochemistry; (**e**), Zip14 immunohistochemistry. Low-power photographs of the stained sections were adjusted for size by reference to the mm scale on the XRF maps and rotated for optimal orientation. The illustrated DRG consisted of two portions of neural tissue that were identified by class-III-β-tubulin (**c**) and Zip14 reaction products (**e**). The region-of-interest containing the bulk of DRG neurons was outlined by interrupted lines. The outline was then transferred to the Zn and Fe XRF maps and the ferritin-stained section. Maps were segmented by vertical and horizontal lines placed at 0.5 mm intervals over the images to generate a grid. Within the maps, each square represented 0.25 mm^2^. At the edges, the squares were smaller. A single Zn or Fe signal was recorded as counts/10 sec from each square, and results of all signals were averaged for each metal. After subtracting background XRF, averaged counts were converted to μg Zn or Fe per ml PEG 1450. In the illustrated case of FA, Zn=4.8 μg/ml and Fe=33.4 μg/ml. The arrows in the two XRF maps indicate the location of Ti wires that are visible on the Zn and Fe XRF maps, respectively, due to minor contamination by these metals. Note strong Zn and Fe XRF arising from the capsule surrounding the neural portions of the DRG. These regions were effectively excluded from quantitative analysis by the illustrated alignment. Bars, 2 mm.

**Figure 2:**
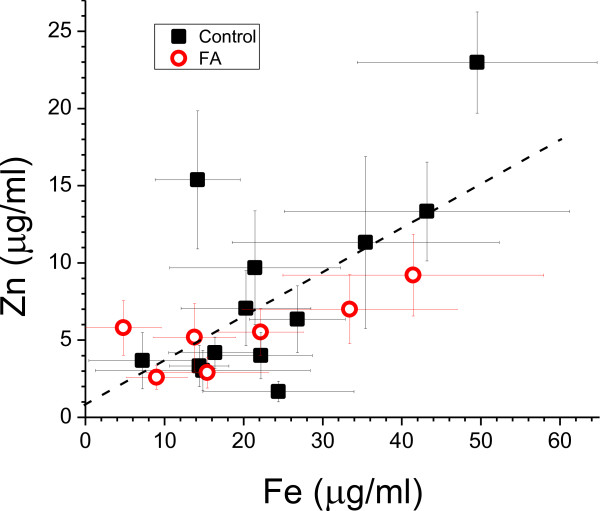
**Correlation of Zn and Fe levels in DRG of 13 normal controls and 7 cases of FA.** Zn levels increase with Fe levels in controls (closed back squares) and FA patients (open red circles). Each symbol represents the mean Zn and Fe level in a single subject, with horizontal and vertical lines representing the S.D. of Zn and Fe determinations, respectively, within that subject. Mean and S.D. are calculated from non-overlapping 0.25 mm^2^ areas in controls and FA, covering the entire neural tissue region of DRG (see methods). Regression result for all subjects is shown by the dashed line: Zn = 0.81 + 0.285 × Fe; slope p<0.001; R^2^=0.45. Multiple linear regression showed no significant difference between controls and FA subjects in either slope (p=0.16) or intercept (p=0.41).

Figure 1 illustrates the alignment of Zn- and Fe-XRF maps with matching sections respectively stained for class-III-β-tubulin, Zip14, and ferritin. Mean concentrations in μg/ml ± S.D. in 13 control subjects were Zn=8.16 ± 6.19, Fe=23.85 ± 12.23. In 7 FA cases, they were Zn = 5.46 ± 2.29 and Fe = 19.99 ± 13.26. The average in each subject was used for a statistical comparison of metal concentrations by t-test which showed no difference between controls and FA. Figure 2 shows a linear regression analysis of Zn and Fe levels in all subjects. Zn and Fe levels are significantly correlated. Multiple linear regressions of control subjects and FA patients disclosed no significant differences of slope or intercept.

### Immunofluorescence and immunohistochemistry

**Figure 3:**
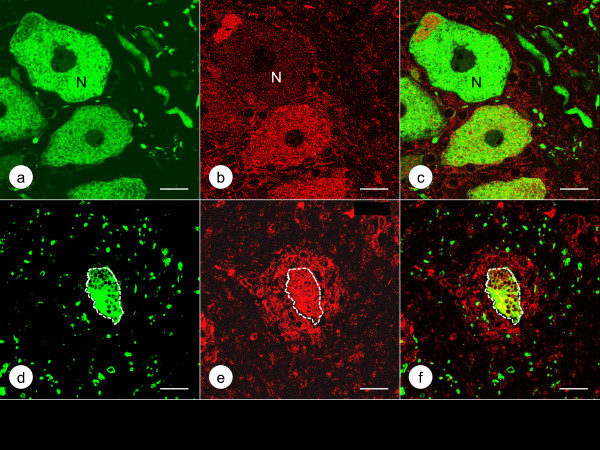
**Double**-**label immunofluorescence of class**-**III**-**β**-**tubulin and Zip14 in a normal control DRG and a DRG of an FA patient.** (**a**)-(**c**) normal control; (**d**)-(**f**) FA; (**a**) and (**d**) class-III-β-tubulin (Alexa488, green); (**b**) and (**e**) Zip14 (Cy3, red); (**c**) and (**f**) merged images. The normal DRG (**a**) shows large neurons that yield intense class-III-β-tubulin reaction product in perikaryon and dendrites. Zip14 staining (**b**) is heterogeneous, with less reaction product in the nerve cell marked by “N”. Anti-Zip14 also visualizes satellite cells (**b**). In FA, a small class-III-β-tubulin-reactive neuron (**d**) continues to display Zip14 reaction product but is surrounded by multiple layers of Zip14-reactive satellite cells (**e**). Bars, 20 μm.

**Figure 4:**
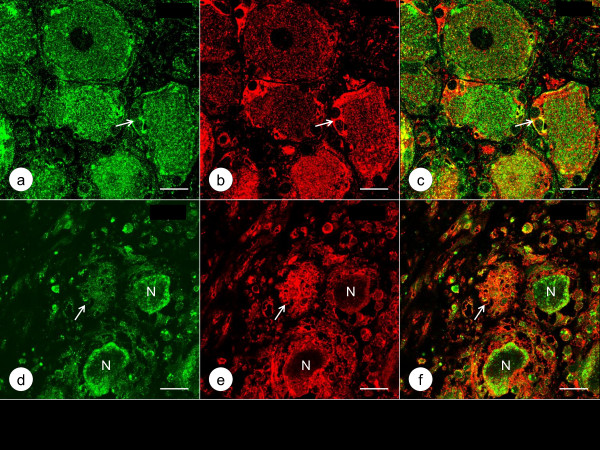
**Double**-**label immunofluorescence of Zip14 and ferritin in a normal control DRG and a DRG of an FA patient.** (**a**)-(**c**) normal control; (**d**)-(**f**) FA; (**a**) and (**d**) Zip14 (Alexa488, green); (**b**) and (**e**) ferritin (Cy3, red); (**c**) and (**f**) merged images. Zip14 (**a**) and ferritin reaction products (**b**) show co-localization in the cytoplasm of several large and small neurons and in perineuronal satellite cells (arrows). Zip14 and ferritin fluorescence in neurons is heterogeneous. In the FA case (**d**-**f**), neuronal Zip14 (**d**) and ferritin (**e**) have shifted to a location beneath the plasma membrane while the central portion of the nerve cells (N) is devoid of reaction product. Zip14 fluorescence is present in satellite cells and a residual nodule (**d**, arrow). Ferritin fluorescence in multi-layer satellite cells and a residual nodule (**e**, arrow) is very prominent. Bars, 20 μm.

**Figure 5:**
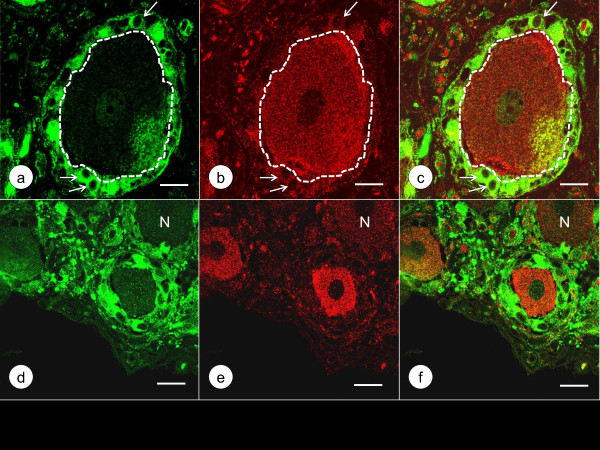
**Double**-**label immunofluorescence of MT1**/**2 and MT3 in a normal control DRG and a DRG of an FA patient.** (**a**)-(**c**) normal control; (**d**)-(**f**) FA; (**a**) and (**d**) MT1/2 (Alexa488, green); (**b**) and (**e**) MT3 (Cy3, red); (**c**) and (**f**) merged images. MT1/2 immunoreactivity is restricted to normal (**a**) and hyperplastic satellite cells in FA (**d**). MT3 immunofluorescence is localized in the cytoplasm of a normal large DRG neuron (**b**) and in satellite cells, in which it co-localizes with MT1/2 (**a-c**, arrows). In FA (**e**), one of three small DRG neurons shows very little MT3 immunofluorescence (N) while two others remain strongly fluorescent. The thickened layers of satellite cells around the 3 neurons show relatively low MT3 fluorescence (**e**). Bars, 20 μm.

Figure 3 shows the localization of class-III-β-tubulin and Zip14 in DRG of a normal control and a case of FA by double-label immunofluorescence. In normal DRG, green class-III-β-tubulin immunofluorescence labels neurons, dendrites, and axons, while red Zip14 fluorescence of variable intensity arises from the cytoplasm of neurons and satellite cells. In FA, class-III-β-tubulin- and Zip14-reactive nerve cells are much smaller, and Zip14 fluorescence is more prominent in multiple layers of satellite cells around atrophic neurons (Figure 3e). Figure 4 presents a comparison of Zip14 and ferritin immunofluorescence. These proteins show extensive co-localization in normal neurons and satellite cells though ferritin immunofluorescence is more intense than Zip14 immunofluorescence in satellite cells. In FA, Zip14- and ferritin-reactive nerve cells are smaller than normal, and the central portions of neurons appear depleted. Ferritin fluorescence arises prominently from multiple layers of satellite cells and a residual nodule (Figure 4e). Figure 5 displays the disparate localization of MT1/2 and MT3 in normal and FA DRG. MT1/2 immunofluorescence arises exclusively from satellite cells. The single perineuronal layer of MT1/2-positive satellite cells in normal DRG (Figure 5a) changes to multiple layers in FA (Figure 5d). In contrast, MT3 immunofluorescence is most prominent in neuronal cytoplasm of normal DRG though low-level emission also arises from satellite cells (Figure 5b). In FA, MT3 immunofluorescence identifies the cytoplasm of small DRG neurons but does not suggest greater expression in hyperplastic satellite cells (Figure 5e).

**Figure 6:**
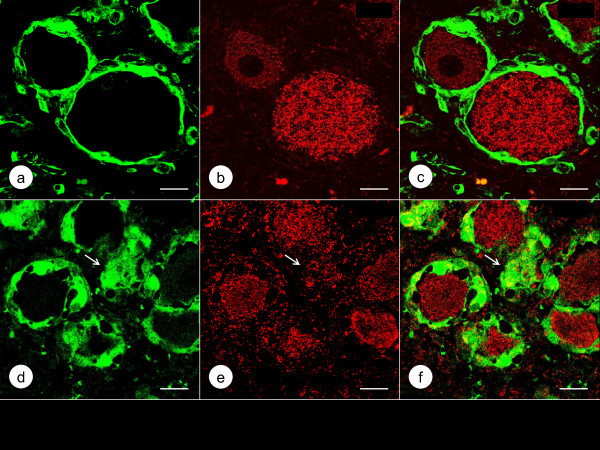
**Double**-**label immunofluorescence of S100α and ATP5B in a normal control DRG and a DRG of an FA patient.** (**a**)-(**c**) normal control; (**d**)-(**f**) FA; (**a**) and (**d**) S100α (Alexa488, green); (**b**) and (**e**) ATP5B (Cy3, red); (**c**) and (**f**) merged images. S100α immunofluorescence is a robust marker of normal satellite cells (**a**) and hyperplastic satellite cells in FA (**d**). In the normal DRG, ATP5B fluorescence is prominent in neuronal cytoplasm (**b**), and only sparse reaction product is present in the tissue between neurons. In contrast, satellite cells in FA (**e**) show a great abundance of granular ATP5B immunofluorescence. The arrows in (**d**)-(**f**) indicate a residual nodule. Bars, 20 μm.

Figure 6 shows intense green immunofluorescence of S100α in satellite cells (Figure 6a) and the great abundance of ATP synthase F1 complex V β-polypeptide (ATP5B)-positive mitochondria (red) in the cytoplasm of normal DRG neurons (Figure 6b). Very little ATP5B fluorescence is present in normal satellite cells (Figure 6b-c). In contrast, the S100α-positive hyperplastic satellite cells and residual nodule in FA display abundant, finely granular, red ATP5B fluorescence (Figure 6e-f).

**Figure 7:**
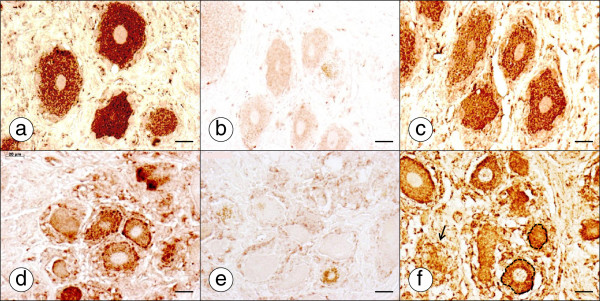
**Frataxin and ATP5B immunohistochemistry of a normal control DRG and a DRG of an FA patient.** (**a**)-(**c**) normal control; (**d**)-(**f**) FA; (**a**) and (**d**) frataxin; (**b**) and (**e**) anti-frataxin antibody pre-absorbed by human recombinant frataxin; (**c**) and (**f**) ATP5B. In the normal DRG, granular frataxin reaction product fills the perikarya of all neurons (**a**). Reaction product in satellite cells is sparse. ATP5B immunohistochemistry yields a similar result (**c**). In FA (**d**-**f**), frataxin reaction product in small neurons is concentrated under the neuronal plasma membrane, and hyperplastic satellite cells display more frataxin-reactive granules than normal (**d**). In FA, ATP5B reaction product shows a distribution in neurons, hyperplastic satellite cells, and a residual nodule that resembles frataxin (**f**, arrow). Two neurons are outlined by interrupted lines. Bars, 20 μm.

Figure 7 shows a comparison of frataxin and ATP5B immunohistochemistry of a DRG in a normal control and a patient with FA. In the normal control, granular frataxin reaction product is present throughout the perikarya of all neurons, with very few reactive granules in satellite cells (Figure 7a). In FA, the antibody reveals smaller neurons, a paucity of granular reaction product in neuronal cytoplasm, and a greater abundance of positive granules in satellite cells (Figure 7d). Pre-absorption of the antibody with recombinant human frataxin eliminates the reaction (Figure 7b and e). Incubation with an antibody to the mitochondrial marker ATP5B yields a very similar result: prominent granular staining of perikarya in normal DRG neurons (Figure 7c) and abundant reaction product in hyperplastic satellite cells in FA (Figure 7f).

## Discussion

### Quantitative in-situ determination of Zn and Fe in DRG

Systematic quantification of Zn in fresh or frozen autopsy specimens of normal DRG has not been reported. Total levels of Fe in DRG of FA patients and normal control subjects, however, are available [2]. Levels were 25.4±10.3 μg/g wet weight (mean ± S.D.) in 3 samples from FA patients and 28±13.4 μg/g wet weight (mean ± S.D.) in 8 normal controls. The difference was not significant. These results are now less applicable because the new method utilizing XRF can restrict measurements to neural tissues of DRG (Figure 1). While the relatively coarse steps (0.15 mm) of the scanning XRF instrument do not resolve the cellular localization of Zn or Fe, non-destructive XRF technology allows for intact tissue samples to be recovered, re-embedded in paraffin, sectioned, and stained for class-III-β-tubulin, Zip14, and ferritin. The critical step is matching XRF metal maps with stained tissue sections. Infiltration by PEG 1000 and PEG 1450 displaces all tissue water, and results expressed as μg metal/ml PEG 1450 (Figure 2) are equivalent to μg metal/ml tissue volume. The results of Zn and Fe can be converted to μg/g wet tissue weight by assuming DRG water content of at least 80 percent. When the mean XRF-recorded Fe levels in normal DRG (23.85 μg/ml PEG) and DRG of FA (19.99 μg/m PEG) are multiplied by 0.8, the result yields 19.08 μg/g wet weight for controls, and 16 μg/g wet weight for FA. These concentrations are lower than the chemical assay of whole DRG [2], reflecting the exclusion of capsule and pericapsular tissues by XRF technology.

### Localization of Zn, Fe, and metal-related proteins in DRG

The normal human DRG may be similar to rat DRG, in which Pérez-Castejón et al. [7] visualized Zn in the cytoplasm of neurons by histochemistry and autoradiography. Velázquez et al. [8] also detected the metal by fluorescence microscopy and MT3 by immunofluorescence in rat DRG. Autolysis due to delayed autopsy imposes severe limitations on the visualization of Zn by chemical and metallographic methods. None of the DRG specimens obtained from the FA patients listed in Table 1 were fixed within the optimal time limit of under 2 h [9,10]. Therefore, this research utilized the immunodetection of Zip14, MT1/2, and MT3 as surrogate markers of Zn, and ferritin as a marker of Fe. The intensity of Zip14 (Figures 3b and 4a) and MT3 immunofluorescence (Figure 5b) suggests that the bulk of Zn in normal DRG is located in the cytoplasm of neurons. In contrast to MT3, the more restrictive immunofluorescence of MT1/2 (Figure 5a and d) implies that this protein provides Zn homeostasis in normal (Figure 5a) and hyperplastic satellite cells (Figure 5d). While ferritin immunofluorescence is stronger in satellite cells than in neurons (Figure 4b), ferritin signal is also present in neurons where it co-localizes with Zip14 (Figure 4a).

### Supportive evidence of Zn and Fe translocation in FA

The described observations establish that FA causes neither influx nor efflux of DRG Zn or Fe. Changes due to FA, however, are evident by immunofluorescence of several metal-handling proteins. The response of Zip14- and ferritin-immunoreactivity to FA is similar, suggesting the transition of Zn and Fe from degenerating neurons to satellite cells occurs by comparable mechanisms. It is likely that MT1/2 reacts to the transfer of Zn from neurons to satellite cells while MT3 remains restricted to smaller neurons (Figure 5e) and presumably disappears when atrophic nerve cells are totally absorbed into residual nodules.

Heretofore, MT3 was thought to be a unique central nervous system (CNS) protein [11], but its presence in DRG (Figure 5b) suggests that neuronal Zn homeostasis is similar.

In CNS, the most prominent Zn transporter is ZnT3, which packages Zn into synaptic vesicles (review in ref [9]). As expected, an antibody to ZnT3 did not generate an immunohistochemical reaction in DRG, which under normal circumstances are devoid of synapses. Zip transporters, including Zip14, are not specific for Zn, and it is noteworthy that the “i” in Zip derives from “iron” [12,13]. Zip14 and MT3 may collaborate in Zn homeostasis of normal DRG neurons. While MT3 is a Zn storage and buffering protein, Zip14 adds Zn transport across plasma membranes. In some DRG neurons of FA, Zip14 immunofluorescence localizes to the cytoplasm just beneath the plasma membrane (Figure 4d). The transmembrane localization of functional Zn transporters may be relevant to this pathological phenomenon.

Origin and physiological role of Zn in normal human DRG are unknown. Velázquez et al. [8] reported that Zn in rat DRG may arise from retrograde axonal transport through dorsal spinal roots. It accumulated only in small-diameter DRG neurons, and the authors [8] considered a role in sensory processing. In humans with FA, loss of Zn-containing DRG neurons may contribute to the complex pathological phenotype observed in dorsal roots [2] and sensory peripheral nerves [14].

### The role of satellite cells in FA

The greater abundance of satellite cells in FA accounts for the hypercellularity of DRG that is readily apparent on routine cell stains [2]. It is likely that the earliest response to the disease is the formation of multiple layers of satellite cells about neurons. This phenomenon is especially apparent by ferritin (Figure 4e) and MT1/2 immunofluorescence (Figure 5d). Residual nodules (Figure 4d-f) are a continuation of satellite cell hyperplasia beyond death of the neuron, but there is no insight yet about the fate of these cellular collections or whether they ultimately shed Zn and Fe into the blood stream.

In normal DRG, neuronal and satellite cell plasma membranes are very closely apposed, with multiple invaginations in both directions [15]. Also, satellite cells are tightly linked to each other. Beyond structural support, they handle “trafficking” into and out of DRG neurons. Pannese [15] also cited experimental studies, in which satellite cells provided “trophic support” to neurons. FA is typically a disease of nerve cells in the central nervous system (CNS) [1], and DRG neurons should therefore be similarly vulnerable to frataxin deficiency. It is a theoretical consideration that hyperplastic satellite cells, which also derive from the neural crest, do not adequately support their immediately adjacent neurons, and neuronal atrophy may be secondary.

The greater abundance of mitochondria in hyperplastic satellite cells and residual nodules in DRG of FA indicates that these cells develop a more active oxidative metabolism as part of the pathological phenotype (Figures 6e and 7f). In normal DRG, satellite cells contain few mitochondria (Figure 6b-c) whereas reaction product of ATP5B is very abundant in the closely apposed neuron. In promptly fixed normal DRG, the available monoclonal anti-frataxin antibody shows reaction product that strongly resembles the distribution of the mitochondrial marker ATP5B (Figure 7). It is apparent that lack of frataxin does not impair the proliferation of satellite cells and the processing of the protein to its mature functional form [16].

### Endogenous metal toxicity or epiphenomenon?

A key question in the pathogenesis of the DRG lesion in FA is: Do Zn and Fe in the cytosol of neurons become toxic, or is the shift in metal-related proteins an epiphenomenon of frataxin deficiency? Human autopsy tissues do not lend themselves to the direct measurement of toxic radicals, but changes in the cellular localization of Zip14, MT1/2, MT3, and ferritin suggest a disturbance is Zn and Fe homeostasis. In the absence of a synaptic source of Zn, the mechanism of toxicity in DRG neurons may be similar to that in CNS neurons of ZnT3-deleted animals [17]. In the cytosol of CNS neurons, ionic Zn must be kept at picomolar or femtomolar levels to prevent toxicity and still meet metabolic demands [18]. It is likely that neurons in normal DRG maintain Zn homeostasis by buffering proteins such as MT3 (Figure 5b) and by sequestration of the metal in mitochondria [18]. The mean level of 8.16 μg/ml in normal DRG, reported in this study, is equal to a concentration of 124.8 μM. Neuronal toxicity is thought to occur at ionic Zn^2+^ levels in the range of 100 nM to 3 μM [18]. Therefore, the total pool of neuronal Zn in DRG is present in large excess over need, and control mechanisms must be very efficient. Dineley et al. [19,20] summarized the evidence of endogenous Zn toxicity in the CNS, in which mitochondria bear the brunt of the damage. Mitochondrial impairment has many untoward effects: insufficient biosynthesis of adenosine triphosphate; decrease of mitochondrial membrane potential; release of cytochrome C and apoptosis-inducing factor; and generation of reactive oxygen species and nitric oxide [18]. All of these processes ultimately cause cell death. If they exist in FA *in vivo*, the untoward effects of Zn on DRG neurons are superimposed on deficient oxidative phosphorylation, which is a recognized effect of frataxin deficiency [4]. Fe in DRG may be less injurious than Zn because holoferritin has a prodigious ability to trap the metal. Therefore, the aggregation of Fe in the shell of ferritin in hyperplastic satellite cells may be protective against Fe-mediated oxidative injury.

Based on experiments with frataxin-deleted mice [21], human neonates with homozygous mutations of the *FXN* gene must have at least some normal frataxin to survive to the mean age of death (40±20 years [N=30; mean ± S.D.]; ref. [22]). It is likely that DRG are hypoplastic at birth in all patients with mutated *FXN* genes [1,2,23]. A superimposed atrophic process may lead to progressive destruction of neurons, principally by satellite cell invasion and absorption into residual nodules. This transition may be the reason why clinical “onset” of FA is delayed to a mean age of 15 years [24]. It may be proposed that in DRG, Zn and Fe dysmetabolism contribute to onset and progression. In contrast to brain, however, nothing is known about the rate by which DRG acquire Zn and Fe during growth. Extrapolating from human brain, it is unlikely that end-point Zn accumulation in DRG coincides with onset of FA [25].

Many questions remain: Does frataxin deficiency trigger endogenous metal toxicity; and why do DRG in FA retain Zn and Fe rather than shedding the metals into the blood stream? The hypothesized accelerated DRG destruction by endogenous metals does not contradict other potential mechanisms of increasing disease activity, such as somatic GAA expansion in postmitotic neurons through the activity of mismatch repair enzymes [26] or greater epigenetic gene silencing [27].

## Conclusions

The neural tissue of DRG contains measurable levels of Zn and Fe. During progressive destruction of DRG in FA, these metals are retained rather than discharged into the circulating blood. Enhanced expression of Zip14, MT1/2, and ferritin in hyperplastic satellite cells of DRG in FA represents indirect evidence that Zn and Fe are lost from degenerating neurons. Metabolic activation of satellite cells in FA is evident by the accumulation of ATP5B-expressing mitochondria. Failing metal homeostasis in DRG neurons may contribute to endogenous Zn toxicity whereas Fe sequestration in holoferritin of hyperplastic satellite cells may be neuroprotective.

## Methods

### Polyethylene glycol embedding of specimens

After fixation in buffered 4% formaldehyde solution for at least 10 days at 4°C, DRG were divided into two portions. One portion was directly embedded in paraffin; the other portion was infiltrated by polyethylene glycol (PEG) 1450 as described before [28]. Tissue samples were immersed at room temperature in aqueous solutions of increasing concentrations (30-90%) of PEG 400 (Sigma, St. Louis, MO, USA), followed by PEG 1000 and PEG 1450 at 60°C. After cooling overnight, PEG 1450 blocks containing DRG tissues were trimmed and “faced” by microtome to present a smooth surface for scanning by XRF. Four segments of titanium (Ti) wire were inserted into the PEG blocks, a short distance from the tissue, to mark the scanning limits of the XRF instrument. Low level Zn and Fe contamination (0.01 percent) in the Ti wires generated Zn and Fe XRF that aided in the subsequent alignment of maps and tissue sections.

### XRF of PEG-embedded DRG and Zn and Fe standards

The custom-built XRF machine consisted of a glass-enclosed chamber housing a molybdenum target X-ray tube, X-ray optics with a doubly-curved crystal, an x-y coordinate scanning mechanism, and a silicon-drift detector [28]. Zn- and Fe-specific XRF was generated with a triple X-ray beam over a period of 10 sec per location with scanning steps of 0.15 mm. The recorded XRF was converted to “maps” of Fe and Zn by the use of a Windows-based proprietary computer program. Pseudocolors indicated relative XRF intensities, with maximum fluorescence as white. Red, orange, green, and light blue represented progressively lower signal strengths. Fe and Zn calibration standards were prepared from Fe-III-mesoporphyrin and Zn-II-mesoporphyrin, respectively, and validated by elemental analysis as described before [28].

### Recovery of DRG from PEG 1450

PEG-embedded DRG tissue was recovered by immersion in phosphate-buffered saline (PBS) and repeated washing in PBS at room temperature (RT). The tissue was post-fixed for several hours at RT in buffered 4% formaldehyde solution (pH 7.2) and embedded in paraffin by routine methods. Six-μm-thick sections were stained by immunohistochemistry with antibodies to class-III-β-tubulin, Zip14, and ferritin as described below.

### Alignment of DRG sections with Zn and Fe maps, and quantitative measurement of Zn and Fe

Low-power photographs (1.25×) of DRG sections stained for class-III-β-tubulin, Zip14, and ferritin were matched to the Zn and Fe XRF maps as shown in Figure 1. The neural tissue of the DRG was outlined based on class-III-β-tubulin and Zip14 reaction product. The limiting outline was transferred to the aligned Zn- and Fe-XRF maps to exclude signals arising from non-neural tissues, and to the ferritin-stained section. Zn and Fe maps were segmented in steps of 0.5 mm by the insertion of vertical and horizontal lines. Each square represented 0.25 mm^2^. Depending on the size of the available tissue sample, this method generated 21–111 squares for the 13 normal DRG and 12–64 squares for the 7 DRG of FA cases. Zn and Fe XRF in each square were recorded as counts/10 sec, and average and standard deviation (S.D.) were determined for each individual. An equal number of locations outside the region defined by the Ti wires were used to determine background XRF. After subtracting background XRF from the Zn- and Fe-signals, counts were converted to μg/ml PEG by reference to calibration standards.

### Antisera, antibodies, antigen retrieval methods, immunohistochemistry, and immunofluorescence

The following antisera (antibodies) were available for immunohistochemistry or immunofluorescence (host species, type of antibody, and commercial source in parentheses): ferritin (rabbit polyclonal, DAKO, Carpinteria, CA, USA; goat polyclonal, GenWay Biotech, San Diego, CA, USA); class-III-β-tubulin (mouse monoclonal antibody TUJ-1, R&D Systems, Minneapolis, MN, USA); Zip14 (rabbit polyclonal, Novus Biological, Littleton, CO, USA); MT1/2 (mouse monoclonal, Invitrogen, Frederick, MD, USA); MT3 (rabbit polyclonal, Sigma-Aldrich, St. Louis, MO, USA); ATP5B (rabbit polyclonal, Santa Cruz Biotechnology, Santa Cruz, CA, USA); S100α (mouse monoclonal, Santa Cruz); frataxin (mouse monoclonal, Mitosciences, Eugene, OR, USA). The MT isoforms 1 and 2 show extensive amino acid homology. The monoclonal antibody reacts with both proteins, hence, the designation “MT1/2”. Optimal antibody dilutions were determined by trial and error, and protein concentrations ranged from 0.2-50 μg protein/ml. Immunohistochemical and immunofluorescence protocols were described in detail in previous publications [2,23,28]. Antigen retrieval methods were DIVA (a proprietary decloaking solution marketed by Biocare Medical, Concord, CA, USA) for 30 min at 95°C for ferritin; incubation in 0.01 M citric acid/sodium citrate buffer (pH 6) for 20 min at 95°C for class-III-β-tubulin, S100α, MT1/2, and MT3; and incubation in 0.1 M tris buffer (pH 9.5) for 20 min at 95°C for ATP5B and frataxin. A chelating step with 2,2′-dipyridyl and sodium hydrosulfite (30 mM each in 0.1 M acetic acid-sodium acetate buffer, pH 4.8) was included for the visualization of ferritin and frataxin. The specificity of frataxin immunohistochemistry was confirmed by preincubation of the antibody solution with a 4-fold excess of recombinant human frataxin (by concentration of protein). Biotinylated secondary antibodies came from Vector Labs (Burlingame, CA, USA). Sigma-Aldrich supplied horseradish peroxidase-labeled streptavidin. Paraffin sections were also used for double-label laser confocal scanning immunofluorescence microscopy of the pairs MT1/2/MT3; Zip14/class-III-β-tubulin; Zip14/ferritin; and S100α/ATP5B. Fluorescently labeled donkey anti-rabbit and anti-mouse IgG antibodies (Alexa488 and Cy3) were purchased from Jackson ImmunoResearch (West Grove, PA, USA). The laser scanning confocal microscope was a Zeiss LSM 510 Meta unit. The exciting wavelengths for Alexa488 and Cy3 were 488 and 543 nm, respectively. Images were obtained at a magnification of 63× under oil immersion and through band pass filters of 500–530 nm for Alexa488 and 565–615 nm for Cy3.

## Abbreviations

ATP5B: ATP synthase F1 complex V β-polypeptide; CNS: Central nervous system; Cu: Copper; DRG: Dorsal root ganglion; FA: Friedreich ataxia; Fe: Iron; FXN: Frataxin gene; GAA: Guanine-adenine-adenine; MT: Metallothionein; PBS: Phosphate-buffered saline; PEG: Polyethylene glycol; RT: Room temperature; SD: Standard deviation; Ti: Titanium; XRF: X-ray fluorescence; Zn: Zinc.

## Competing interests

The authors declare that they have no competing interest.

## Authors’ contribution

AHK designed the study, drafted the manuscript, and assembled the illustrations. ECK, STB, and RLR provided the laboratory techniques, including PEG infiltration, XRF, tissue recovery from PEG, immunohistochemistry, and immunofluorescence. JEM contributed double-label laser scanning confocal immunofluorescence microscopy. PJF performed the statistical analysis of quantitative data and prepared Figure 2. All authors studied and revised the manuscript, and agreed on a final version prior to submission.
